# The Temperature in Ruptured Ectopic Gestation Sac

**Published:** 1905-09-09

**Authors:** 


### THE TEMPERATURE IN RUPTURED ECTOPIC GESTATION SAC.

The diagnosis of rupture of an ectopic gestation sac is a matter which is frequently attended with difficulty. Nevertheless, it is essential in the interest of the patient that a mistake should not be made. The commonest errors, perhaps, are to mistake rupture of an ectopic gestation sac for ordinary abortion and vice versa. The results in either case may be lamentable. It is worth while, therefore, to consider any points which may assist the medical man to make a correct diagnosis in any doubtful case. Such a help is to be found, according to Dr. S. A. Brickner,[1] in the patient's temperature. In abortion the temperature, as a rule, is not raised above the normal level. But in ectopic pregnancy, when rupture of the sac has occurred, the patient's temperature is usually found to be elevated. Thus in thirty cases which Dr. Brickner has studied, the temperature was taken previously to operation in 22, and of these 19, or 86 per cent., had a temperature above 99° F. These observations are quite in accordance with general experience. For blood in the peritoneal cavity, whatever may be the reason of its presence in that situation, is commonly accompanied by an increase of the body temperature. But the fact is not always appreciated at the bedside; and it is for this reason that we call attention to the matter, and quote Dr. Brickner's figures. The medical attendant in a case of supposed abortion should be sufficiently alert to observe, and to regard with suspicion, an elevation of the patient's temperature; and in a case of suspected rupture of an ectopic pregnancy, a normal temperature in the absence of marked collapse should be recognised as unusual, and as calling for a suspension of opinion upon the diagnosis until every avenue of clinical inquiry has been explored.

[1] Medical News, Aug. 12, 1905.

